# Light and polyphosphate kinase 2 cooperatively regulate the production of zero-valent sulfur in a deep-sea bacterium

**DOI:** 10.1128/msystems.00473-25

**Published:** 2025-05-16

**Authors:** Tianhang Zhang, Ruining Cai, Chaomin Sun

**Affiliations:** 1 CAS and Shandong Province Key Laboratory of Experimental Marine Biology & Center of Deep Sea Research, Institute of Oceanology, Chinese Academy of Sciences 53014, Qingdao, China; 2 Laboratory for Marine Biology and Biotechnology, Qingdao Marine Science and Technology Center, Qingdao, China; 3 College of Earth Science, University of Chinese Academy of Sciences, Beijing, China; 4 Center of Ocean Mega-Science, Chinese Academy of Sciences, Qingdao, China; University of Florida, Gainesville, Florida, USA

**Keywords:** infrared light, polyphosphate kinase 2, zero-valent sulfur, c-di-GMP, deep-sea bacterium

## Abstract

It is well established that different wavelengths of light exist in various deep-sea environments, and many deep-sea microorganisms have evolved specialized mechanisms for sensing and utilizing light energy. Our previous research found that blue light promotes zero-valent sulfur (ZVS) production in *Erythrobacter flavus* 21-3, a bacterium isolated from a deep-sea cold seep. Given that long-wavelength light is more prevalent in deep-sea environments, the present study investigates the mechanism by which *E. flavus* 21-3 senses infrared light (wavelength 940 nm) and regulates ZVS production. We found that the bacteriophytochrome BPHP-15570 is responsible for sensing infrared light, which induces autophosphorylation of BPHP-15570, activating the diguanylate cyclase DGC-0450 for c-di-GMP biosynthesis. Subsequently, the PilZ domain-containing protein mPilZ-1753 binds to c-di-GMP, triggering a well-established ZVS production pathway involving thiosulfate dehydrogenase (TsdA) and two homologs of thiosulfohydrolases (SoxB). Notably, polyphosphate kinase 2 (PPK2) is recruited to compete for GTP, the direct precursor of c-di-GMP biosynthesis. This competition downregulates ZVS production as well as other important metabolic processes. This negative regulatory pathway helps the bacterium avoid excessive ZVS accumulation, which could be toxic to bacterial growth. Overall, *E. flavus* 21-3 has evolved a sophisticated regulatory pathway to sense both blue and infrared light, triggering ZVS production. Our study provides a valuable model for understanding light utilization and its coupling with sulfur cycling in deep-sea environments.

**IMPORTANCE:**

It is widely believed that deep-sea ecosystems operate independently of light, relying primarily on chemical energy. However, the discovery of non-photosynthetic bacteria in various deep-sea environments that can sense and utilize light has challenged this assumption. In a recent study, we found that blue light significantly promotes the production of zero-valent sulfur (ZVS) in the deep-sea bacterium *Erythrobacter flavus* 21-3. Given that long-wavelength light is more prevalent in deep-sea environments, we investigated whether infrared light also plays a role in regulating ZVS production in *E. flavus* 21-3. Our results indicate that infrared light does promote ZVS formation in this bacterium. We identified PPK2 as a negative regulator, maintaining intracellular ZVS at safe levels to prevent toxicity due to excessive accumulation. Overall, our study offers a valuable model for exploring how light is utilized and its interaction with microbial sulfur cycling in the extreme conditions of the deep sea.

## INTRODUCTION

Light is a rich and diverse source of energy and information, making it unsurprising that many microorganisms have evolved the ability to sense and harness it (1). Based on their photosynthetic abilities, photosensitive bacteria are classified into two groups: photosynthetic bacteria and non-photosynthetic bacteria (2, 3). Non-photosynthetic bacteria utilize light to generate signals that activate specific cellular functions, leading to an output response (4). These bacteria are believed to possess photosensitive proteins (5). The chromophore, an auxiliary factor for these proteins, plays a crucial role in capturing light stimuli, with different chromophores being sensitive to specific wavelengths of light (1). Chromophores are linked to receptor proteins through either covalent or non-covalent bonds. Upon absorbing photons, they trigger signal transduction, influencing the organism’s biological processes (6).

Among the photosensitive proteins, those that have been extensively studied include bacteriophytochrome (BPHP), light-oxygen-voltage (LOV) proteins, riboflavin-based blue light receptors (BLUF), and photosensitive yellow proteins (PYP) (7–11). Many bacteria respond to light through a two-component system that includes LOV histidine kinases (LOV HKs) and their response regulators (12–17). Upon activation, LOV HKs modulate the effector domains of their response regulators through transphosphorylation, thereby influencing gene expression and various cellular functions (12, 14, 16, 18). Bacteriophytochromes, a group of photoreceptors, are particularly significant for microorganisms as they are the most abundant and widespread light-sensing photoreceptors in bacteria (19). The binding of different bilins (linear tetrapyrroles) and their binding modes enable bacteriophytochromes from various organisms to detect changes in light conditions (20, 21). All bilins are derived from heme molecules through the action of heme oxygenase (22). Bacteriophytochromes undergo reversible light-induced conversion under red and infrared light. Their N-terminal photoreceptor region consists of three domains: PAS (Per/Arnt/Sim), GAF (cGMP phosphodiesterase/adenyl cyclase/FhlA), and PHY (phytochrome), with the GAF domain containing chromophore binding sites. The C-terminal light regulatory region includes a histidine kinase (HK) domain that facilitates signal transduction (21, 23).

It is noteworthy that many non-photosynthetic bacteria, isolated from various deep-sea environments, have been found to sense and utilize light through distinct photosensitive proteins. For instance, *Spongiibacter nanhainus* CSC3.9, a bacterium isolated from a deep-sea cold seep, can sense blue light via a BLUF protein, thereby regulating its motility (24). Similarly, *Croceicoccus marinus* OT19, a bacterium isolated from deep-sea hydrothermal vents, can detect infrared light (with a wavelength of 940 nm) to enhance its growth through a bacteriophytochrome photoreceptor (22). Numerous studies have confirmed that light regulates various metabolic processes in bacteria, including bacterial adhesion, biofilm formation and disintegration, virulence, pigment synthesis, and flagellar movement (25–27).

Zero-valent sulfur (ZVS) is common beneath the seafloor, especially in cold seeps and hydrothermal systems (28–31). It plays a key role in energy conservation for prokaryotes in these environments. ZVS, closely associated with microbial cells, serves as a bio-signature of sulfur-oxidizing microorganisms (32–34). Its production begins with polysulfide formation, which is crucial in geochemical processes like pyritization (34). Cyclooctasulfur (S_8_) and inorganic polysulfide (S_*n*_^2−^) are important ZVS forms, serving as vital intermediates in the sulfur cycle (35). In a recent study, we found that blue light significantly promotes the production of ZVS in the deep-sea bacterium *Erythrobacter flavus* 21-3 (3). The light–oxygen–voltage (LOV) histidine kinase LOV-1477 responds to blue light and activates the diguanylate cyclase (DGC-2902), which produces c-di-GMP. This, in turn, allows the PilZ domain-containing protein mPilZ-1753 to bind to c-di-GMP and activate thiosulfate dehydrogenase (TsdA) through direct interaction. TsdA, along with two homologs of thiosulfohydrolases (SoxB), cooperate to produce ZVS (Fig. S1) (3). This finding highlights an intriguing link between blue light sensing and ZVS production. However, we still have two unsolved concerns that need to be addressed: (i) whether infrared light regulates ZVS production in *E. flavus* 21-3, considering that longer wavelengths may be more prevalent in deep-sea environments, and (ii) how *E. flavus* 21-3 negatively regulates ZVS production, given that excessive ZVS can be toxic to bacterial cells (36, 37).

In the present study, we find that, in addition to blue light, infrared light also significantly promotes the formation of zero-valent sulfur (ZVS) in *E. flavus* 21-3. Using a combination of proteomic, genetic, and biochemical approaches, we show that the bacteriophytochrome (BPHP-15570) senses infrared light and activates the downstream diguanylate cyclase (DGC-0450), leading to c-di-GMP biosynthesis. This molecule, in turn, triggers the thiosulfate oxidation pathway involving TsdA and SoxB for ZVS production. Finally, we identify polyphosphate kinase 2 (PPK2) as a novel negative regulator that maintains intracellular ZVS levels at a safe threshold, preventing toxicity caused by excessive ZVS accumulation.

## RESULTS

### Infrared light promotes the production of ZVS in a deep-sea bacterium

**Fig 1:**
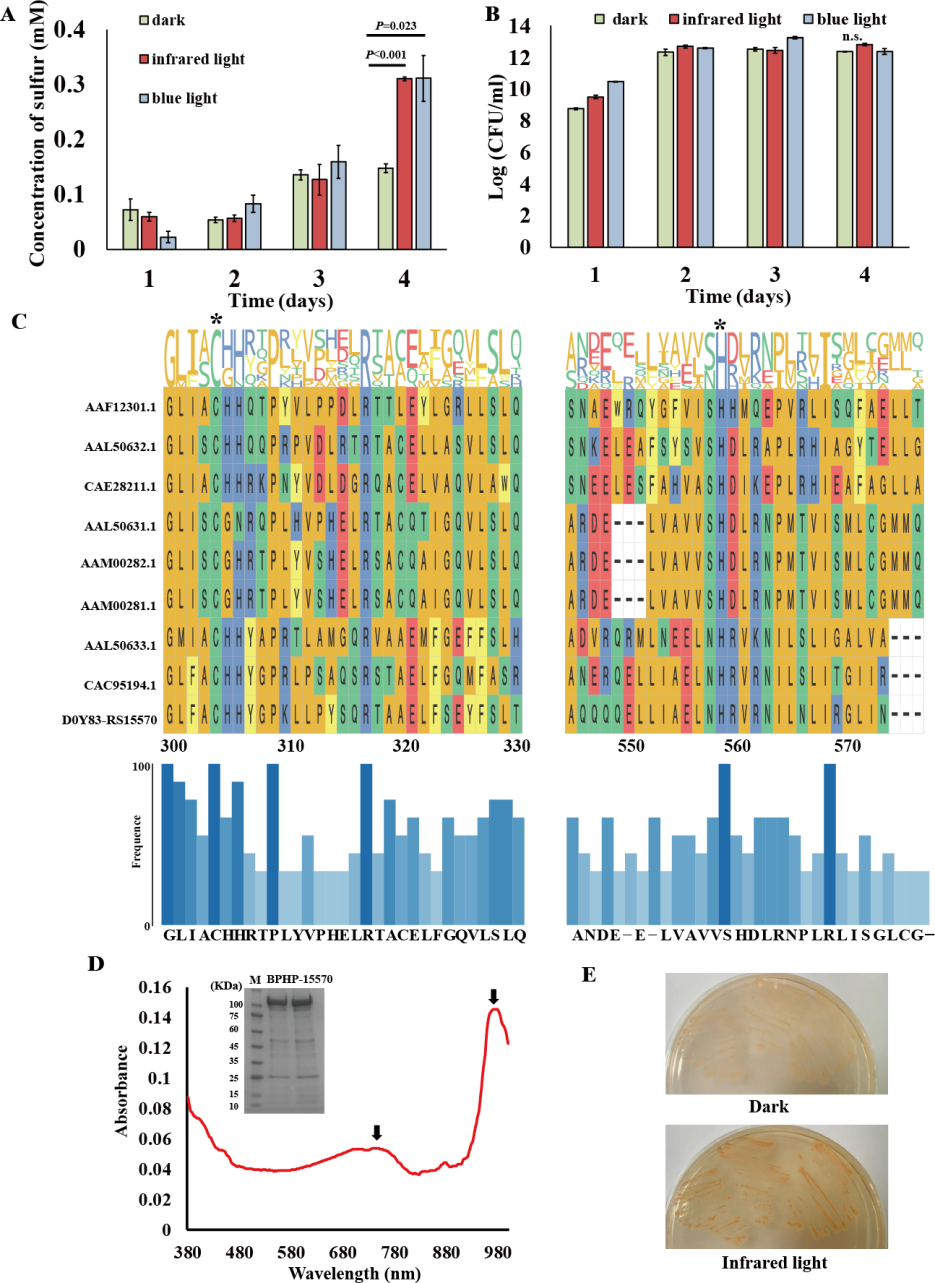
A bacteriophytochrome (BPHP-15570) responds to infrared light and triggers the production of ZVS in the deep-sea bacterium *E. flavus* 21-3. (**A**) Comparison of ZVS production in *E. flavus* 21-3 cultured under blue light, infrared light, or dark conditions for 1, 2, 3, and 4 days (*N* = 3 biological replicates). (**B**) Comparison of the growth of *E. flavus* 21-3 cultured under blue light, infrared light, or dark conditions for 1, 2, 3, and 4 days (*N* = 3 biological replicates; n.s. indicates no significant difference). (**C**) Sequence alignment of D0Y83-RS15570 and other homologs. The conserved binding sites (Cys in the GAF domain and His in the HK domain) are marked with asterisks. The numbers on the left indicate the accession numbers of the corresponding proteins. The blue bar chart shows the frequency of base occurrence. (**D**) Absorption spectrum analysis of BPHP-15570 after infrared light illumination. Black arrows indicate the absorption peaks corresponding to infrared light. The inset shows the SDS-PAGE analysis of purified BPHP-15570 from *E. coli* BL21 (DE3) cells (M, protein marker). (**E**) Infrared light sensing assay of BPHP-15570 in *E. coli* BL21 (DE3) cells. *E. coli* BL21 (DE3) cells co-expressing BPHP-15570 and heme oxygenase displayed the red color on Congo red-containing agar plates (50 µg/mL) under infrared light illumination.

In our previous studies, *E. flavus* 21-3 was shown to produce zero-valent sulfur (ZVS) in the form of S_8_ through a novel thiosulfate oxidation pathway, both in laboratory (38) and deep-sea *in situ* (39) conditions. Interestingly, we discovered that blue light significantly promoted the production of ZVS (3). Considering the dim environment of the deep sea, infrared light might be more prevalent. To investigate this, we compared the yields of ZVS in *E. flavus* 21-3 under blue light, infrared light, and dark conditions. The results indicated that after 4 days of cultivation, ZVS production was significantly higher under infrared light compared to the dark condition, and the levels were similar to those observed under blue light (Fig. 1A). To rule out the possibility that the differences in ZVS production were due to variations in biomass, we compared the biomass and ZVS yield of bacterial cells grown under infrared light and dark conditions. Colony-forming unit (CFU) results showed no significant differences in growth between *E. flavus* 21-3 cultured under infrared light and dark conditions (Fig. 1B). Therefore, we concluded that the differences in ZVS yields were not related to growth discrepancies between the experimental conditions.

### A bacteriophytochrome (BPHP-15570) responds to infrared light and triggers the production of ZVS

To investigate how *E. flavus* 21-3 senses infrared light and activates the production of ZVS, we searched for proteins that possess the potential to sense infrared light in the genome of *E. flavus* 21-3. Generally, bacteriophytochrome is proposed to sense infrared light (22). With that, we searched proteins annotated as bacteriophytochrome in the genome of *E. flavus* 21-3. The search results identified two candidates, bacteriophytochrome D0Y83-RS15570 and D0Y83-RS09925, in *E. flavus* 21-3 (Fig. S2A). Both possess the GAF domain, which is photosensitive and commonly found in various red and infrared light photoreceptors (23). However, D0Y83-RS09925 lacks the PAS domain, preventing it from performing a photosensitive function (Fig. S2A). In addition, previous studies indicate that bacteriophytochromes require binding to the corresponding chromophore, bilins, which are synthesized by heme oxygenase (HO), to function properly (22). Notably, a gene encoding heme oxygenase is located immediately upstream of the gene for D0Y83-RS15570 (Fig. S2B). Therefore, we hypothesize that D0Y83-RS15570 may function as the infrared light photoreceptor in *E. flavus* 21-3.

To verify whether D0Y83-RS15570 functions as a bacteriophytochrome, we first predicted its structural domains using SMART. The results revealed that it contains key bacteriophytochrome domains, including PAS and GAF (Fig. S2A). Notably, D0Y83-RS15570 also features a histidine kinase (HK) domain and a REC domain, suggesting its potential for autophosphorylation and activation of downstream signaling pathways upon light stimulation (14, 21). Further analysis confirmed the presence of essential conserved amino acids—Cys in the GAF domain, His in the HK domain, and Asp in the REC domain—critical for the function of these domains within bacteriophytochromes (Fig. 1C; Fig. S3). Taken together, the sequence characteristics of D0Y83-RS15570 align well with known bacteriophytochrome structures (20, 40). Therefore, we designated it as BPHP-15570 in this study.

To investigate the photo-sensing and spectral properties of BPHP-15570, we co-expressed BPHP-15570 with its upstream heme oxygenase (HO) in the *E. coli* BL21 (DE3) cell line, as HO is essential for bacteriophytochrome to perform its photosensing function. Spectrophotometric analysis revealed that the purified BPHP-15570 exhibited two main absorption peaks at approximately 780 nm and 970 nm (Fig. 1D), which are characteristic of bacteriophytochromes. Interestingly, *E. coli* cells co-expressing BPHP-15570 and HO displayed the red color on a Congo red-containing agar plate when illuminated with infrared light (Fig. 1E). According to previous reports (41), when the curly fimbriae of *E. coli* bind Congo red, the color changes to red, and this fimbriae formation is closely associated with intracellular c-di-GMP concentration. These results suggest that BPHP-15570 can sense infrared light in *E. coli* cells and trigger the production of c-di-GMP. To further confirm its infrared light-sensing capability *in vivo*, we attempted to delete the BPHP-15570 gene from the genome of *E. flavus* 21-3 using recombination methods. However, despite extensive efforts, we were unable to delete the gene, indicating that BPHP-15570 is likely essential for the growth of *E. flavus* 21-3. Taken together, these findings suggest that BPHP-15570 plays a critical role in responding to infrared light and triggering the production of ZVS in *E. flavus* 21-3.

### The bacteriophytochrome BPHP-15570 activates the diguanylate cyclase DGC-0450 for c-di-GMP biosynthesis

**Fig 2:**
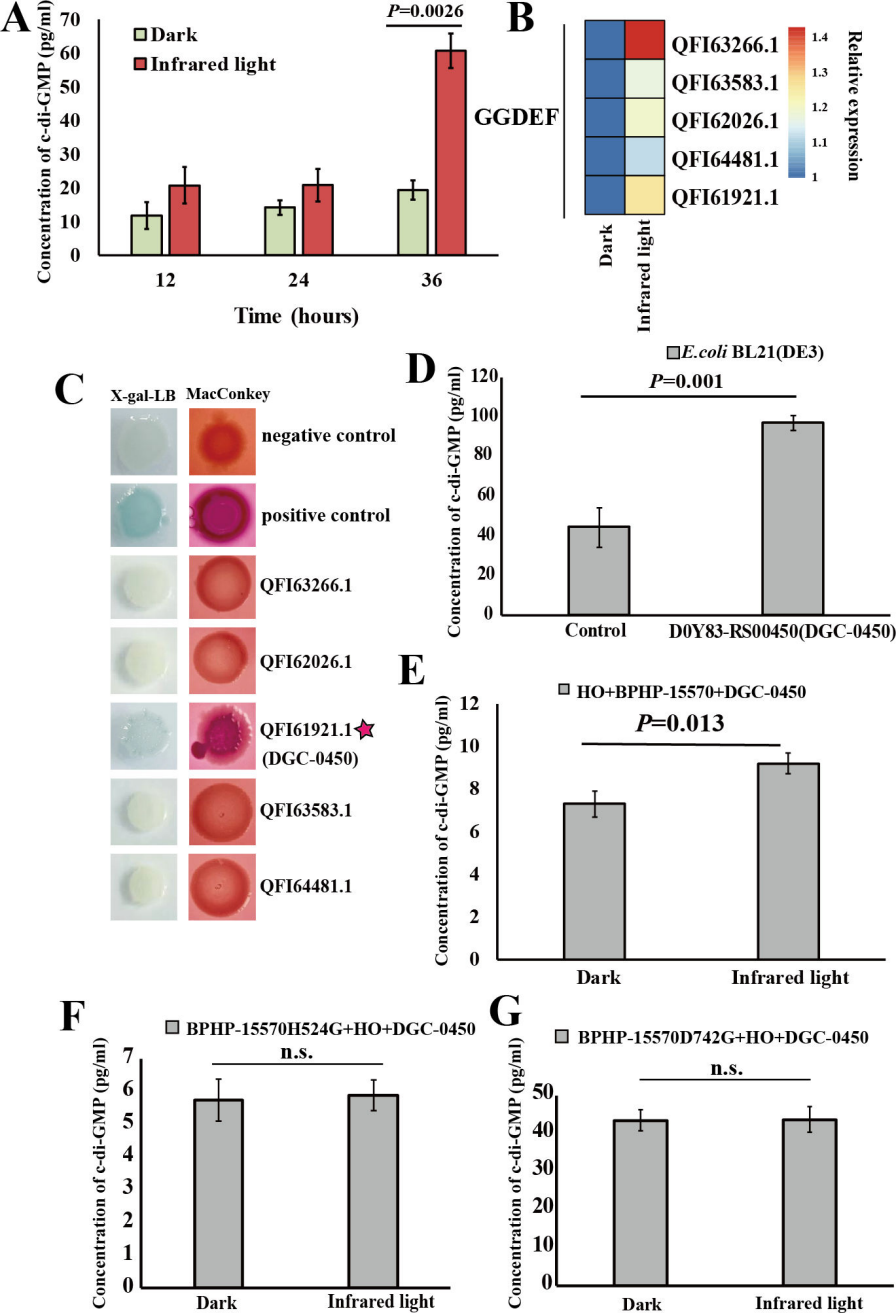
The bacteriophytochrome BPHP-15570 activates the diguanylate cyclase DGC-0450 for c-di-GMP biosynthesis. (**A**) Comparison of c-di-GMP yields in *E. flavus* 21-3 cultured under infrared light and dark conditions for 12, 24, and 36 hours (*N* = 3 biological replicates). (**B**) Proteomic analysis revealed the expression levels of five GGDEF-domain-containing proteins in *E. flavus* 21-3, which were significantly up-regulated under infrared light compared to dark conditions. (**C**) Detection of interaction between BPHP-15570 and the predicted GGDEF-domain-containing proteins. Assays were performed on MacConkey plates with 1% maltose and X-gal-LB indicator plates (LB broth with 40 µg/mL X-gal) with 0.5 mM IPTG. *E. coli* BTH101 strains were transformed with T18 and T25 fusion constructs to exclude self-activation. Positive control: *E. coli* BTH101 with pKNT25-Zip and pCH363-Zip plasmids. Negative control: *E. coli* BTH101 with empty plasmids. (**D**) Comparison of intracellular c-di-GMP concentrations in *E. coli* BL21(DE3) with and without the overexpression of DGC-0450. The c-di-GMP concentration in *E. coli* BL21(DE3) transformed with the empty vector was normalized as the control (*N* = 3 biological replicates). (**E**) Comparison of intracellular c-di-GMP concentrations in *E. coli* BL21(DE3) co-expressing heme oxygenase (HO), BPHP-15570, and DGC-0450 under infrared light and dark conditions (*N* = 3 biological replicates). (**F**) Comparison of intracellular c-di-GMP concentrations in *E. coli* BL21(DE3) co-expressing BPHP-15570 mutant (with His524 mutated to Gly) and heme oxygenase (HO) after 24 hours of cultivation under infrared light and dark conditions (*N* = 3 biological replicates; n.s. indicates no significant difference). (**G**) Comparison of intracellular c-di-GMP concentrations in *E. coli* BL21(DE3) co-expressing BPHP-15570 mutant (with Asp742 mutated to Gly) and heme oxygenase (HO) after 24 hours of cultivation under infrared light and dark conditions (*N* = 3 biological replicates; n.s. indicates no significant difference).

Building on our previous findings, the light-oxygen-voltage histidine kinase (LOV-1477) of *E. flavus* 21-3 responds to blue light and activates the diguanylate cyclase DGC-2902 to produce c-di-GMP, which drives ZVS production in *E. flavus* 21-3 (3). Notably, the key domains (HK domain and REC domain) of the two-component system for blue light sensing are located separately in the light sensor LOV-1477 and the diguanylate cyclase DGC-2902, whereas the infrared light sensor BPHP-15570 contains both the HK and REC domains (Fig. S2A). Given the differences between blue light and infrared light sensors, we next aimed to investigate whether c-di-GMP also plays a critical role in regulating ZVS production under infrared light conditions. To test this, we measured the intracellular c-di-GMP content in *E. flavus* 21-3 exposed to infrared light. The results showed that after 36 hours of incubation, the c-di-GMP levels in *E. flavus* 21-3 under infrared light were significantly higher than those under dark conditions (Fig. 2A), mirroring the pattern of ZVS production observed under infrared light and dark conditions (Fig. 1A). These findings suggest that c-di-GMP indeed plays a key role in driving ZVS production under infrared light. Therefore, we propose that the infrared light sensor BPHP-15570 interacts with the downstream diguanylate cyclase to trigger c-di-GMP biosynthesis and regulate ZVS production (42). To better find the potential diguanylate cyclase that interacts with BPHP-15570, we conducted a proteomic analysis of *E. flavus* 21-3 cultured under both infrared light and dark conditions for 72 hours. Proteomic analysis revealed that the expression levels of five GGDEF domain-containing proteins in *E. flavus* 21-3 were higher under infrared light than in dark conditions (Fig. 2B; Fig. S4). This suggests that these five GGDEF domain-containing proteins may serve as interaction partners of BPHP-15570. Previous research results indicate that the GGDEF domains are involved in the turnover of cyclic-di-GMP (c-di-GMP) *in vivo* and stimulate c-di-GMP production (42). Subsequently, to verify whether BPHP-15570 interacts with these five proteins, we conducted the bacterial two-hybrid experiment. Briefly, assays were done on MacConkey with 1% maltose and the X-gal-LB indicator plate (LB broth with 40 µg/mL X-gal) with 0.5 mM IPTG. Reporter strains of *E. coli* BTH101 were transformed, respectively, using T18 and T25 fusion constructs to exclude self-activation. The *E. coli* BTH101 strain harboring pKNT25-Zip and pCH363-Zip plasmids was used as the positive control (3). Through bacterial two-hybrid experiments, we identified that D0Y83-RS00450 likely interacts with BPHP-15570 (Fig. 2C). To confirm whether D0Y83-RS00450 is a diguanylate cyclase capable of generating c-di-GMP, we overexpressed it in *E. coli* BL21 (DE3). Subsequently, we measured the intracellular c-di-GMP content in *E. coli* BL21 (DE3) cells, and the results showed that the c-di-GMP concentration in cells expressing D0Y83-RS00450 was significantly higher than that in the control group (Fig. 2D). These findings confirm that D0Y83-RS00450 is indeed a diguanylate cyclase, which we have named DGC-0450 in this study.

It is well established that the histidine (His) within the HK domain and the aspartate (Asp) within the response regulator (REC) domain are critical for the function of two-component systems (14, 16, 18). Since both the HK and REC domains are located within BPHP-15570, we hypothesize that infrared light triggers autophosphorylation of BPHP-15570, leading to the activation of the downstream diguanylate cyclase DGC-0450. This activation subsequently results in the production of c-di-GMP as an output response.

**Fig 3:**
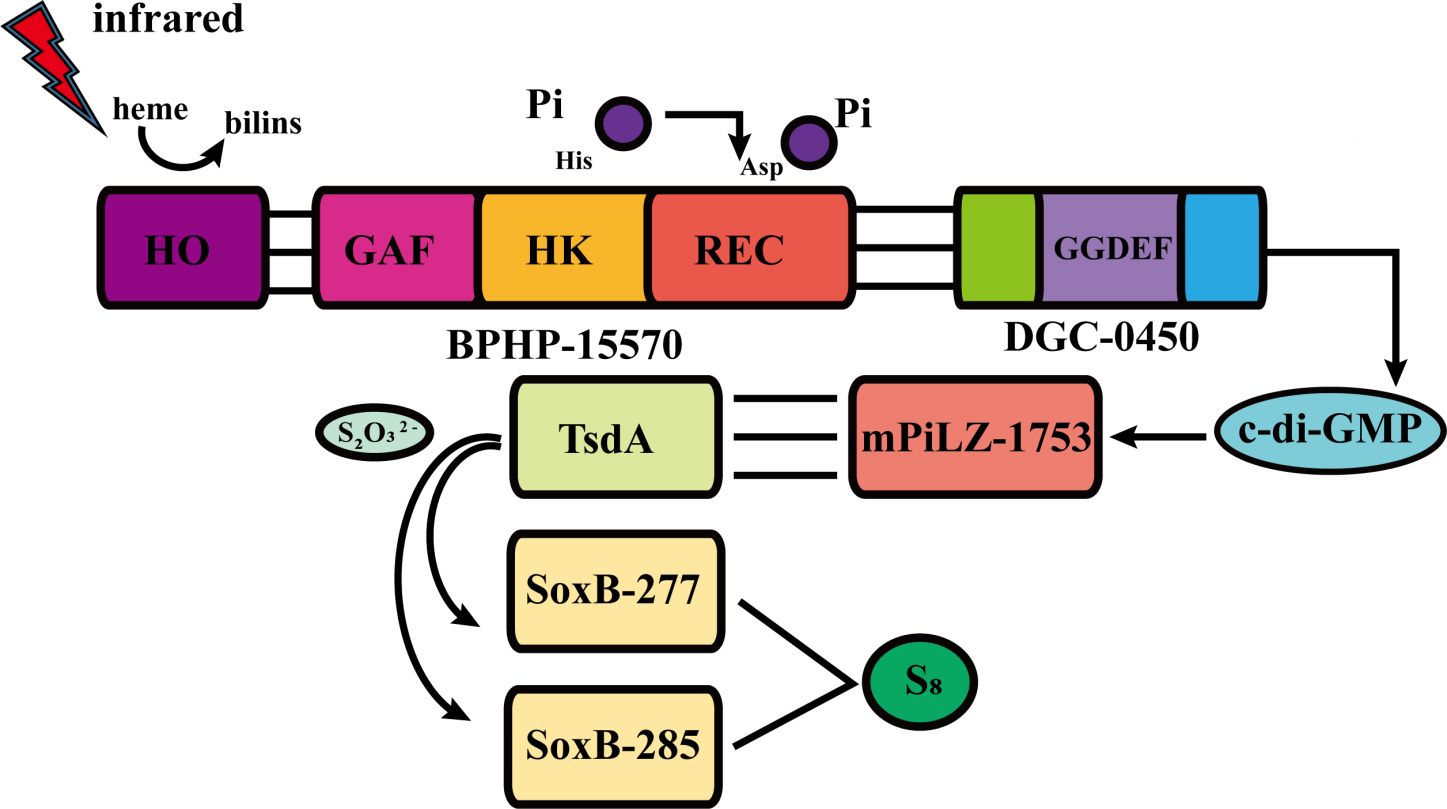
Proposed model for ZVS production pathway triggered by infrared light in *E. flavus* 21-3. In the presence of infrared light, BPHP-15570 senses the light signal with the assistance of heme oxygenase (HO), which induces autophosphorylation and activates the diguanylate cyclase DGC-0450, leading to the production of c-di-GMP. The generated c-di-GMP then binds to mPilZ-1753, triggering its interaction with thiosulfate dehydrogenase (TsdA). This interaction facilitates the conversion of thiosulfate to tetrathionate. Finally, tetrathionate is further transformed into ZVS by thiosulfohydrolases SoxB-277 and SoxB-285. HO: heme oxygenase; TsdA: thiosulfate dehydrogenase; SoxB: thiosulfohydrolase; PPK2: polyphosphate kinase 2.

To test this hypothesis, we co-expressed HO-BPHP-15570 with DGC-0450 in *E. coli* BL21 (DE3) cells and examined their coordinated functions. As anticipated, we observed a higher concentration of c-di-GMP in *E. coli* BL21 (DE3) cells exposed to infrared light compared to those grown in the dark (Fig. 2E). By contrast, when the conserved 524His residue (Fig. 2F) or the conserved 742Asp residue (Fig. 2G) in BPHP-15570 were mutated to glycine, no significant differences in c-di-GMP concentrations were observed in *E. coli* BL21 (DE3) cells grown under infrared light and in the dark. Taken together, these results suggest that upon infrared light stimulation of *E. flavus* 21-3, BPHP-15570 undergoes autophosphorylation, activating the downstream diguanylate cyclase DGC-0450, which results in the increased production of c-di-GMP. As previously discovered (3), c-di-GMP binds to mPilZ-1753, which then activates TsdA through direct interaction. This leads to the conversion of more thiosulfates to tetrathionates. Finally, tetrathionates are ultimately transformed into ZVS by SoxB277 and SoxB-285 (Fig. 3).

### Polyphosphate kinase 2 (PPK2) downregulates the production of ZVS and formation of biofilm by decreasing the biosynthesis of c-di-GMP

**Fig 4:**
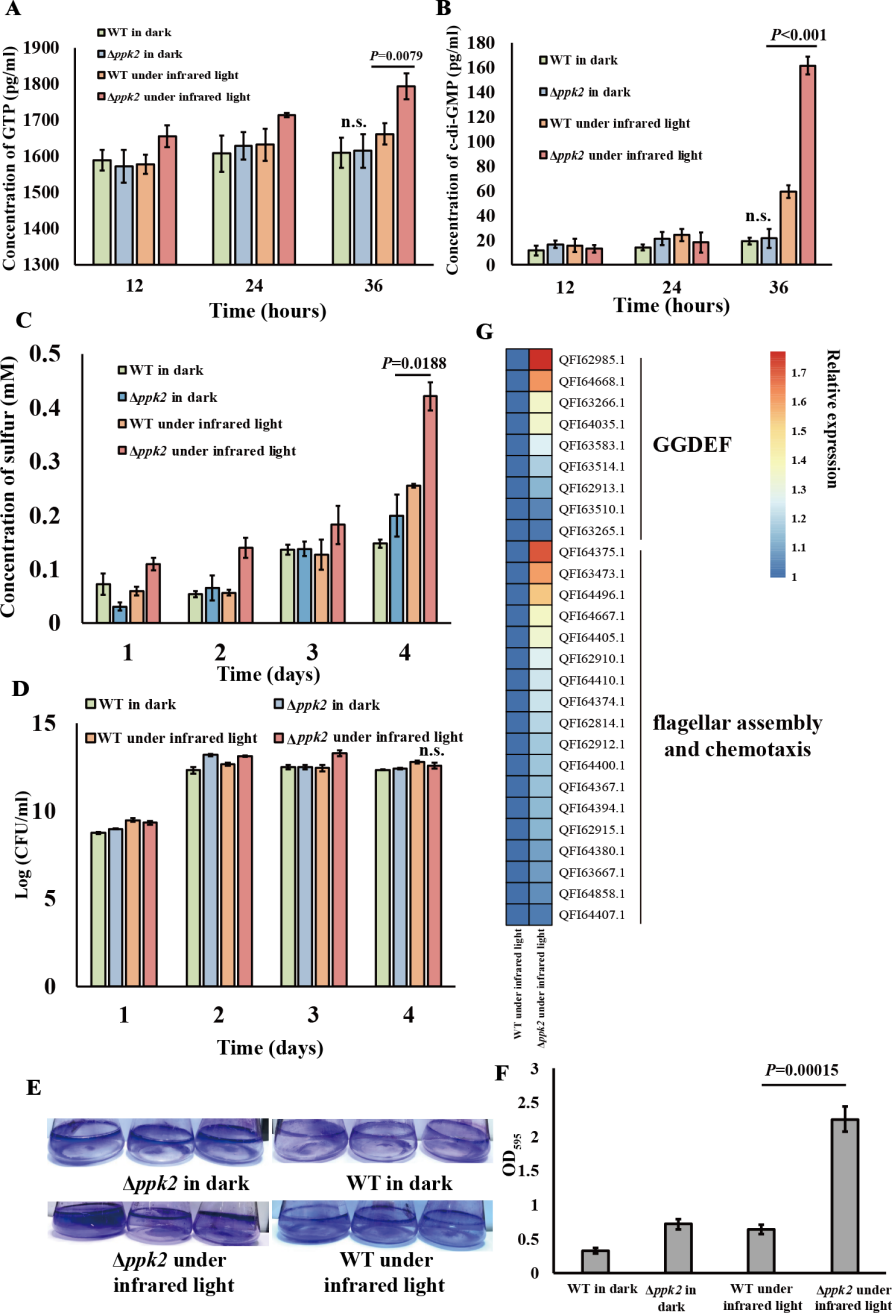
Downregulation of ZVS formation by polyphosphate kinase 2 (PPK2) through decreased c-di-GMP biosynthesis. (**A**) Comparison of intracellular GTP concentrations in wild-type and mutant Δ*ppk2* strains of *E. flavus* 21-3 cultured under infrared light and dark conditions for 12, 24, and 36 hours (*N* = 3 biological replicates; n.s. indicates no significant difference). (**B**) Comparison of intracellular c-di-GMP concentrations in wild-type and mutant Δ*ppk2* strains of *E. flavus* 21-3 cultured under infrared light and dark conditions for 12, 24, and 36 hours (*N* = 3 biological replicates; n.s. indicates no significant difference). (**C**) Comparison of ZVS concentrations produced by wild-type and mutant Δ*ppk2* strains of *E. flavus* 21-3 cultured under infrared light and dark conditions for 1, 2, 3, and 4 days (*N* = 3 biological replicates). (**D**) Comparison of growth levels of wild-type and mutant Δ*ppk2* strains of *E. flavus* 21-3 cultured under infrared light and dark conditions for 1, 2, 3, and 4 days (*N* = 3 biological replicates; n.s. indicates no significant difference). (**E**) Qualitative analysis of biofilm formation in wild-type and mutant Δ*ppk2* strains of *E. flavus* 21-3 cultured under infrared light and dark conditions (*N* = 3 biological replicates). (**F**) Quantitative analysis of biofilm formation as shown in panel E. (**G**) Proteomic analysis of the expression of GGDEF-domain-containing proteins, as well as flagellar assembly and chemotaxis-related proteins, in wild-type and mutant Δ*ppk2* strains of *E. flavus* 21-3 cultured under infrared light.

The accumulation of ZVS in the bacterial periplasm can be toxic (36, 37). How does *E. flavus* 21-3 regulate ZVS production to prevent such harmful effects? Our previous and current findings clearly demonstrate that c-di-GMP is a crucial factor in regulating ZVS production in the deep-sea bacterium *E. flavus* 21-3. As a result, we are focused on identifying a protein in *E. flavus* 21-3 that can negatively regulate c-di-GMP levels, inhibiting its synthesis and consequently reducing ZVS accumulation. c-di-GMP is synthesized from two GTP molecules by diguanylate cyclase (DGC) in microorganisms (43). On the other hand, polyphosphate kinase 2 (PPK2) requires ATP or GTP to carry out its function and exerts similar effects to c-di-GMP in bacteria (44). PPK2 was first identified in a *Pseudomonas aeruginosa* null mutant lacking *ppk1* and is distinguished from PPK1 by its ability to synthesize polyphosphate (polyP) using either GTP or ATP (44). Increasingly, PPK2 is recognized as a key regulatory factor in bacterial physiology and virulence, contributing to processes such as bacterial homeostasis, stress response, biofilm formation, cell invasion, and antibiotic resistance (44). Based on this, we speculate that PPK2 may compete with c-di-GMP for GTP utilization, ultimately leading to a reduction in c-di-GMP concentration. To test this hypothesis, we identified a gene (D0Y83-RS04100) encoding PPK2 in the *E. flavus* 21-3 genome, and deleted the *ppk2* gene to construct the *ppk2* knockout strain of *E. flavus* 21-3. We next measured the intracellular GTP levels in both the wild-type strain and the Δ*ppk2* mutant under infrared light and dark conditions. The results revealed that under infrared light, the Δ*ppk2* mutant exhibited significantly higher GTP production than the wild-type strain after 36 hours of cultivation, while no significant difference was observed under dark conditions (Fig. 4A). Next, we assessed the intracellular c-di-GMP content in both strains under the same light conditions. We found that, under infrared light, the Δ*ppk2* mutant had significantly higher c-di-GMP concentrations compared to the wild-type strain after 36 hours of cultivation (Fig. 4B). By contrast, no significant difference was detected under dark conditions, suggesting that PPK2 acts as a negative regulator of c-di-GMP production specifically under infrared light.

Upon exposure to infrared light, the Δ*ppk2* mutant showed a significantly higher concentration of ZVS than the wild-type strain of *E. flavus* 21-3 after 4 days of incubation (Fig. 4C). However, under dark conditions, no significant difference in ZVS concentration was observed between the mutant and wild-type strains. To determine whether this difference was due to variations in biomass between the Δ*ppk2* mutant and wild-type strain under infrared light, we measured the biomass of both strains at different growth stages. The results indicated no significant biomass differences between the wild-type and mutant strains under both dark and infrared light conditions up to 4 days of incubation (Fig. 4D). These findings suggest that the increased ZVS production in the Δ*ppk2* mutant is likely due to the loss of PPK2 function, which results in the accumulation of c-di-GMP, promoting more ZVS formation. In addition, we observed that the Δ*ppk2* mutant produced more biofilm than the wild-type strain under infrared light (Fig. 4E and F), further indicating that PPK2 regulates biofilm formation in *E. flavus* 21-3 by modulating c-di-GMP biosynthesis. Consistently, the proteomic results showed that the expression levels of some GGDEF-domain-containing proteins, related to flagellar assembly and chemotaxis, as well as proteins with biofilm formation potential, were higher than the control group (Fig. 4G). Taken together, we proposed that PPK2 acts as a negative regulator of ZVS and biofilm formation in *E. flavus* 21-3 by downregulating c-di-GMP production.

**Fig 5:**
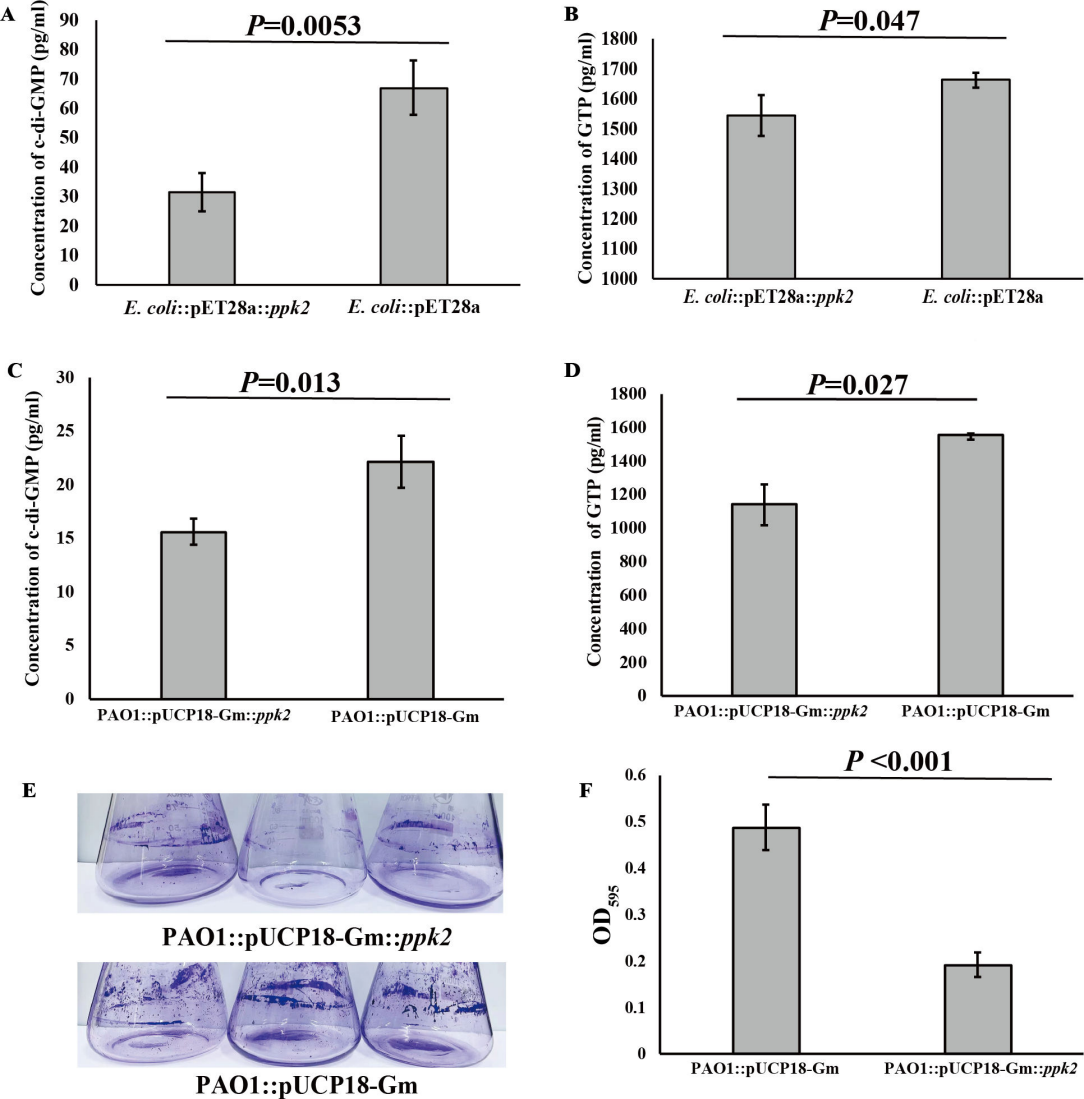
Downregulation of c-di-GMP production by polyphosphate kinase 2 (PPK2) in *E. coli* BL21 (DE3) and *P. aeruginosa* PAO1. (**A**) Comparison of intracellular c-di-GMP concentrations in *E. coli* BL21 (DE3) overexpressing PPK2 and the control. The control strain consists of *E. coli* BL21 (DE3) overexpressing the empty vector pET28a (+) (*N* = 3 biological replicates). (**B**) Comparison of intracellular GTP concentrations in *E. coli* BL21 (DE3) overexpressing PPK2 and the control. The control strain consists of *E. coli* BL21 (DE3) overexpressing the empty vector pET28a (+) (*N* = 3 biological replicates). (**C**) Comparison of intracellular c-di-GMP concentrations in *P. aeruginosa* PAO1 overexpressing PPK2 and the control. The control strain consists of *P. aeruginosa* PAO1 overexpressing the empty vector pUCP18-Gm (*N* = 3 biological replicates). (**D**) Comparison of intracellular GTP concentrations in *P. aeruginosa* PAO1 overexpressing PPK2 and the control. The control strain consists of *P. aeruginosa* PAO1 overexpressing the empty vector pUCP18-Gm (*N* = 3 biological replicates). (**E**) Qualitative analysis of biofilm formation in *P. aeruginosa* PAO1 overexpressing PPK2 and the control. The control strain consists of *P. aeruginosa* PAO1 overexpressing the empty vector pUCP18-Gm. (**F**) Quantitative analysis of biofilm formation as shown in panel E (*N* = 3 biological replicates).

To determine whether PPK2 functions in other bacteria, we overexpressed *ppk2* in the *E. coli* BL21 (DE3) cell line and measured intracellular c-di-GMP and GTP concentrations. The results revealed that both c-di-GMP and GTP levels were significantly lower in the overexpressing strain compared to the control group (Fig. 5A and B), indicating that PPK2 negatively regulates c-di-GMP production by competing for GTP utilization. Furthermore, c-di-GMP has been shown to regulate biofilm formation in *Pseudomonas aeruginosa* PAO1, where high intracellular c-di-GMP levels promote biofilm formation, while low levels increase cell motility and trigger biofilm dispersal (45). To investigate whether PPK2 also influences biofilm formation in *P. aeruginosa* PAO1 through modulation of c-di-GMP and GTP concentrations, we expressed *ppk2* in *P. aeruginosa* PAO1 and measured intracellular c-di-GMP and GTP levels. The results showed significantly lower levels of both c-di-GMP (Fig. 5C) and GTP (Fig. 5D) in the *ppk2*-expressing strain, along with a marked reduction in biofilm biomass compared to the control group (Fig. 5E and F).

The results clearly indicate that PPK2 acts as a negative regulator of various metabolic pathways, such as ZVS production and biofilm formation, in the deep-sea bacterium *E. flavus* 21-3 and other bacteria (*E. coli*, *P. aeruginosa* PAO1), by inhibiting the biosynthesis of c-di-GMP.

## DISCUSSION

In the present study, we demonstrate that light and polyphosphate kinase 2 (PPK2) collaboratively regulate ZVS production in the deep-sea bacterium *E. flavus* 21-3. To our knowledge, this is the first report showing that a deep-sea bacterium can sense both blue and infrared light to modulate its ZVS production. Elemental sulfur, serving as a crucial intermediate metabolite in the sulfur cycle (46), is widely present in deep-sea environments (28), particularly in cold seeps and hydrothermal ecosystems (29, 31). Under natural conditions, elemental sulfur reacts with hydrogen sulfide ions to form polysulfide compounds (S_*n*_^2-^, where *n* = 2–9) (47). The chain length of polysulfides is closely related to the environmental pH, with longer chains forming at higher pH values; near neutral conditions, polysulfides tend to form cyclic S_8_ (48). The sulfur atoms in the intermediate portions of elemental sulfur and polysulfides are collectively referred to as zero-valent sulfur (ZVS) (48–50). During microbial metabolism of sulfide or thiosulfate, a mixture of elemental sulfur and polysulfides is typically formed, existing in the form of sulfur globules (46, 51). In previous studies, it was found that *E. flavus* 21-3 can utilize ZVS produced by itself as a nutrient for growth (39). The production and secretion of ZVS is an energy-saving strategy for *E. flavus* 21-3 to adapt to the deep-sea cold seeps. They utilize abundant thiosulfate as nutrition, converting it into ZVS. Then, ZVS is transported outside the cells and subsequently serves as their energy reserve. During periods of nutrient scarcity, *E. flavus* 21-3 will attach to and utilize ZVS as an electron donor to promote better growth (39).

Traditionally, the deep sea was thought to be devoid of light, and it was widely believed to be a typical chemical ecosystem independent of light (52). However, accumulating evidence has revealed that the deep ocean contains two forms of light: geological light and bioluminescence produced by luminous organisms (3). This light can range from short-wavelength light (e.g., blue light) to long-wavelength light (e.g., infrared light) (53). As a result, deep-sea microorganisms have evolved diverse mechanisms to sense and utilize light. In 2005, an obligate photosynthetic anaerobe was isolated from a deep-sea hydrothermal vent, capable of photosynthetic growth under extremely low light intensities (54). Similarly, the first representative of *Candidatus* Thermofonsia Clade 2 within *Chloroflexi* was cultured and shown to follow a typical phototrophic lifestyle both in the laboratory and deep-sea conditions (55). Beyond photosynthetic microbes, non-photosynthetic microorganisms have also been found to utilize light. For example, the deep-sea bacterium *Croceicoccus marinus* OT19, isolated from hydrothermal vents, can sense infrared light through bacteriophytochrome and enhance growth via light-mediated metabolic pathways (22). Another deep-sea bacterium, *Idomarina* sp. OT37-5b, isolated from hydrothermal sediments in the Okinawa Trough, generates CdS nanoparticles and captures light energy through them to promote growth (56). These findings collectively challenge the long-held belief in deep-sea darkness, highlighting the crucial role of light in the survival and metabolism of deep-sea microorganisms. In the harsh and energy-scarce deep-sea environment, microorganisms that can harness even weak light gain a competitive advantage.

**Fig 6:**
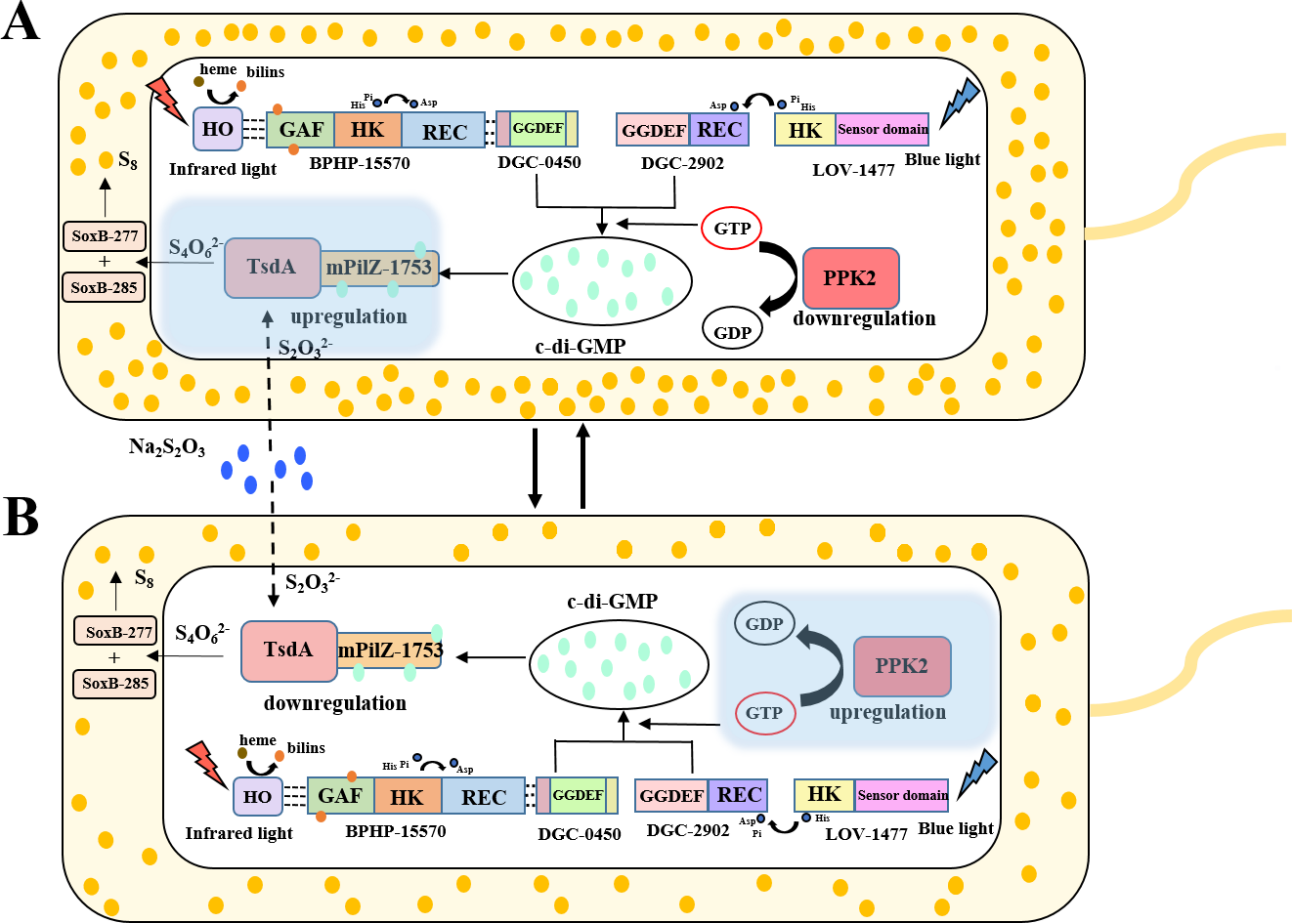
Proposed model for the cooperative regulation of ZVS production by light and polyphosphate kinase in *E. flavus* 21-3. (**A**) In the presence of light (infrared or blue) and thiosulfate, *E. flavus* 21-3 activates light sensors BPHP-15570 and LOV-1477, which, respectively, detect infrared and blue light. These light sensors then interact with the diguanylate cyclases DGC-0450 and DGC-2902, stimulating their activity to produce c-di-GMP. Concurrently, the expression of PPK2 is negatively regulated. As a result, c-di-GMP accumulates and binds to mPilZ-1753, promoting the ZVS production pathway via TsdA and SoxB. (**B**) To prevent the harmful effects of excessive ZVS production, *E. flavus* 21-3 increases the expression of PPK2, which consumes intracellular GTP and reduces c-di-GMP generation. This, in turn, downregulates the downstream production of ZVS. HO: heme oxygenase; TsdA: thiosulfate dehydrogenase; SoxB: thiosulfohydrolase; PPK2: polyphosphate kinase 2.

Combining our previous and present studies (3), we clearly demonstrate that both blue and infrared light promote the ZVS production in the deep-sea bacterium *E. flavus* 21-3. And both light-sulfur coupling pathways involve two-component regulatory systems (TCRSs). The core mechanism of TCRSs consists of phosphotransfer between two conserved components: a histidine kinase (HK) and a response regulator (RR). HK serves as the “input” component, sensing external stimuli and regulating the signal transduction pathway accordingly. RR, as the “output” component, receives the regulation from HK and modulates downstream signaling. Upon sensing a signal, HK undergoes autophosphorylation on its histidine residue, generating a high-energy phosphoryl group, which is then transferred to the aspartate residue of RR. This phosphorylation induces a conformational change in RR, activating it and triggering the corresponding response (16). In the presence of blue light, the histidine kinase LOV-1477 activates its cognate response regulator, the diguanylate cyclase (DGC-2902), to initiate c-di-GMP biosynthesis, which subsequently triggers the downstream ZVS production pathway. Upon exposure to infrared light, the bacteriophytochrome BPHP-15570 is activated to sense the light signal, leading to autophosphorylation of both the HK and REC domains. This activation stimulates the diguanylate cyclase DGC-0450, resulting in the production of c-di-GMP, which also directs the same ZVS production pathway. Clearly, c-di-GMP plays a crucial role in regulating the ZVS production pathway under both blue and infrared light in *E. flavus* 21-3. As a widely recognized bacterial second messenger, c-di-GMP is synthesized from two GTP molecules through the action of diguanylate cyclases (57). c-di-GMP plays a pivotal role in regulating various biological functions, including cell differentiation, biofilm formation, virulence factors, and bioluminescence (57, 58). In this study, we demonstrate that c-di-GMP is a key regulator of light utilization and ZVS formation in *E. flavus* 21-3. Our results suggest that c-di-GMP acts as a metabolic regulatory switch in deep-sea microorganisms, an aspect that should not be overlooked. We also found that polyphosphate kinase 2 (PPK2) reduces ZVS yield in *E. flavus* 21-3 by downregulating c-di-GMP levels. In bacteria, inorganic polyphosphate (polyP) is synthesized by polyphosphate kinase (PPK) enzymes, which are generally classified into two families: PPK1 and PPK2 (59, 60). PPK1, first isolated from *E. coli*, reversibly catalyzes the reversible synthesis of polyP by transferring the terminal phosphate from ATP (61). PPK2 is distinguished from PPK1 by its ability to synthesize polyP using either GTP or ATP (61, 62). However, in this study, PPK2 is found to regulate the generation of ZVS, as well as the formation of biofilm and chemotaxis, by affecting the content of c-di-GMP in *E. flavus* 21-3. Therefore, PPK2 is believed to play a negative feedback regulatory role in *E. flavus* 21-3. Based on these findings, we propose a model describing the interplay between light and polyphosphate kinase 2 in regulating ZVS production in *E. flavus* 21-3 under deep-sea conditions. When the bacterium is exposed to light (either infrared or blue) and thiosulfate, the light sensors BPHP-15570 or LOV-1477 detect the light, activating the diguanylate cyclases DGC-0450 or DGC-2902 to produce c-di-GMP. During this stage, PPK2 expression is downregulated (Fig. S5), allowing for a high c-di-GMP concentration that activates mPilZ-1753, thereby triggering the ZVS production pathway regulated by TsdA and SoxB (Fig. 6A). However, excessive accumulation of ZVS in the bacterial periplasm can be toxic (36, 37). To prevent the harmful effects of overproduction, the bacterium upregulates PPK2 expression (Fig. S5), which sequesters intracellular GTP, leading to decreased c-di-GMP levels and reduced ZVS production (Fig. 6B). This strategy allows the bacterium to efficiently utilize light as a resource for ZVS synthesis while controlling the amount of ZVS produced to prevent cellular damage. In conclusion, our study provides a comprehensive model for understanding the coupling of light utilization with microbial sulfur cycling in deep-sea environments.

## MATERIALS AND METHODS

### Bacterial strains and cultivation conditions

*Escherichia coli* strains for vector construction were grown in Luria Bertani (LB) broth (1% tryptone, 0.5% yeast extract, and 1% NaCl in deionized water) at 37°C. *Erythrobacter flavus* 21-3 and its mutant strains were cultured in 2216E broth (0.5% tryptone and 0.1% yeast extract in seawater) at 28°C, with the pH adjusted to 7.2–7.5 using 2 M NaOH. The cultures of *E. flavus* 21-3 exhibited high turbidity after ZVS formation, which interfered with accurate OD_600_ measurements for bacterial growth. Consequently, the growth status of *E. flavus* 21-3 was assessed using the CFU method. Briefly, the culture was serially diluted, spread onto 2216E agar plates, and colonies were counted after 3 days of incubation.

### Determination of the amount of ZVS in the bacterial cultures

To determine the concentration of ZVS produced by *E. flavus* 21-3 and its mutants, bacterial cultures were grown at 28°C in 2216E broth supplemented with 40 mM sodium thiosulfate. ZVS was then extracted from the culture using trichloromethane, following a previously described method (63). Briefly, 2 mL of the sample was extracted twice with 5 mL of trichloromethane. The extracts were subsequently measured using a UV-Vis spectrometer (Infinite M1000 Pro; Tecan, Männedorf, Switzerland) at a wavelength of 270 nm. To assess the ZVS concentration generated by the *E. flavus* 21-3 strains under various light conditions, bacteria were cultivated in 2216E broth with 40 mM sodium thiosulfate for 3 days, under different light exposures or in darkness. The light exposure conditions included blue light (wavelengths of 465–470 nm [20 µmol m^−2^s^−1^]), infrared light (wavelengths of 940 nm [5 µmol m^−2^s^−1^]), and complete darkness.

### Protein structure prediction and annotation

To predict the structure of BPHP-15570, SMART was employed with default parameters (64). Protein sequences were retrieved in FASTA format from the Uniprot database. The motifs and conserved amino acids were identified by aligning multiple sequences against known sequences using MAFFT (65). The resulting data were visualized using R packages, including msa, ggmsa, Biostrings, ggseqlogo, and cowplot (66–68).

### Cloning, overexpression, and purification of heme oxygenase (HO) and bacteriophytochrome BPHP-15570

Heme oxygenase (HO) is essential for the synthesis of bilins, the chromophore of bacteriophytochrome. Due to the autocatalytic binding of the chromophore to apo-bacteriophytochromes, functional recombinant bacteriophytochrome complexes can be easily obtained by co-expressing the genes encoding bacteriophytochrome and heme oxygenase in *E. coli*. To achieve this, the genes *bphp-15570* (2,547 bp) and *ho* (558 bp) were amplified via PCR using the genomic DNA of *E. flavus* 21-3 and primers listed in Table S1. The thermal cycling conditions were as follows: an initial denaturation step at 94°C for 10 minutes, followed by 30 cycles of amplification (94°C for 45 seconds, 50°C for 45 seconds, 72°C for 90 seconds), and a final extension at 72°C for 10 minutes. The PCR products of *bphp-15570* and *ho* were gel-purified, then cloned into the pET28a (+) vector and pGEX4T-1 vector, respectively, via homologous recombination. After transforming into competent *E. coli* DH5α cells, positive recombinants were selected on LB-agar plates containing kanamycin (50 µg/mL) and ampicillin (100 µg/mL), and further confirmed by sequencing. The correct recombinant plasmids (pET28a (+)/BPHP-15570 and pGEX4T-1/HO) were then transformed into the expression host *E. coli* BL21 (DE3) cells, which were cultured in 1 L of LB broth at 37°C, supplemented with 1 mL kanamycin (50 µg/mL) and ampicillin (100 µg/mL). When the culture reached an OD_600_ of 0.6–0.8, IPTG was added to a final concentration of 0.2 mM, and the culture was incubated further at 16°C for 16 hours.

The bilin-bound BPHP-15570 was purified following previously described protocols, with all procedures conducted at temperatures below 4°C (69). Briefly, cell lysates prepared by sonication were centrifuged at 8,000 *× g* for 20 minutes at 4°C. The supernatant was collected and loaded onto a HisTrap HP column (GE Healthcare, USA), where the His-tagged target protein was eluted using 300 mM imidazole in 10 mM Tris–HCl buffer (pH 8.0). The imidazole was subsequently removed by dialysis. The dialyzed solution was then applied sequentially to a HiTrap Q HP column (GE Healthcare, USA) and a HiLoad 16/600 Superdex 200 column (GE Healthcare, USA). The pure active fractions were collected and stored for further analysis.

To assess infrared light-dependent curli fimbriae production, *E. coli* cells co-expressing HO and BPHP-15570 were cultured on LB agar plates under both dark and infrared light conditions. The LB agar medium was supplemented with Congo red and 0.5 mM IPTG. The production of curli fimbriae, which bind Congo red, was monitored to evaluate the effect of infrared light (41).

### Proteomic analysis

Proteomic analysis was performed by PTM-Bio Labs (Hangzhou, China). Briefly, *E. flavus* 21-3 and its mutant strain were cultured at 28°C in 2216E medium supplemented with 40 mM sodium thiosulfate, under both infrared light and dark conditions. After 72 hours, cells were harvested by centrifugation at 5,000 *× g* for 10 minutes at 4°C. Protein samples were digested, labeled, separated, and quantified using LC-MS/MS (liquid chromatography-mass spectrometry). Bioinformatics analyses, including protein annotation, functional classification, functional enrichment, and clustering, were performed. All MS data have been deposited in the ProteomeXchange Consortium via the PRIDE partner repository (70). Detailed experimental methods are provided in the Supplemental material.

### Construction of gene deletion mutants of *E. flavus* 21-3

To delete the *bphp-15570* and *ppk2* genes from the *E. flavus* 21-3 genome, the upstream and downstream flanking regions of these genes were amplified using the primers listed in Table S2. The PCR products were purified, combined, and used as templates for overlap extension PCR. The resulting PCR products were purified, digested, and inserted into the suicide vector pEX18-Gm. The recombinant vector was then sequentially transformed into *E. coli* SY327 and *E. coli* S17-1. Bacterial conjugation between the *E. flavus* 21-3 strain and *E. coli* S17-1, containing the recombinant vector, was performed by culturing in 2216E broth at 28°C for 3 days. The conjugated bacteria were then resuspended and incubated in 2216E broth for 2 hours at 28°C with shaking at 150 rpm. The strains were selected on 2216E agar plates supplemented with 25 µg/mL gentamicin and 100 µg/mL ampicillin. After 5 days of cultivation, the selected strains were further cultured in 2216E broth for 48 hours and then plated again on 2216E agar plates containing 10% sucrose. Putative mutants were verified using primers listed in Table S2.

### Determination of the concentration of c-di-GMP

According to the previous protocol (3), 50 mL cultures of *E. flavus* 21-3, *E. coli* BL21 (DE3), and *P. aeruginosa* PAO1 were collected by centrifugation at 3,000 *× g* for 10 minutes. The supernatant was discarded, and the cell pellets were resuspended in 40 mL phosphate-buffered saline (PBS). The resuspended pellets were then sonicated, and the remaining debris was removed by centrifugation at 10,000 *× g* for 10 minutes at 4°C. The resulting supernatant was used to determine the concentration of c-di-GMP using the c-di-GMP ELISA kit (Meimian, China).

### Bacterial two-hybrid assay

As described previously (3), the T25 and T18 fragments are used in the two-hybrid system and are complementary parts of the adenylate cyclase (CyaA) catalytic domain. Briefly, the two potential interacting proteins are fused to the T25 and T18 fragments, respectively, and then co-expressed in the *E. coli* BTH101 strain, which lacks CyaA. When these proteins interact, functional complementation between T25 and T18 occurs, activating CyaA and resulting in cAMP production. The cAMP binds to the catabolite activator protein (CAP), forming the cAMP/CAP complex. In *E. coli* BTH101, the cAMP/CAP complex positively regulates the expression of the lacZ gene (encoding β-galactosidase), producing blue colonies on agar plates. The cAMP/CAP complex can also activate the transcription of maltose genes, leading to red colonies on maltose/phenol red indicator plates upon acidification (3). For bacterial adenylate cyclase two-hybrid assays, recombinant pKNT25 and pCH363 plasmids carrying the target genes were used in various combinations and co-transformed into *E. coli* BTH101 cells. The transformed cells were incubated at 30°C with shaking at 150 rpm for 6 hours. Subsequently, 2 µL of the culture was plated onto LB-X-gal-IPTG medium (1% NaCl, 1% tryptone, 0.5% yeast extract, 1.5% agar, 0.5 mM IPTG, 40 µg/mL X-gal, and 50 µg/mL streptomycin) and MacConkey medium (4% MacConkey agar, 0.5 mM IPTG, 50 µg/mL streptomycin, and 1% maltose), followed by incubation at 30°C for 48 hours. The primers used in this experiment are listed in Table S3.

### Detection and measurement of biofilm formation in *E. flavus* 21-3 and *Pseudomonas aeruginosa* PAO1

For the biofilm formation assay, 500 µL of *E. flavus* 21-3 or *P. aeruginosa* PAO1 culture was inoculated into conical flasks containing 50 mL of liquid 2216E or LB medium. The flasks were incubated at 28°C (for *E. flavus* 21-3) or 37°C (for *P. aeruginosa* PAO1) without shaking for further biofilm analysis. To quantify biofilm formation, the liquid medium and planktonic cells were removed from the conical flasks, and the remaining biofilm was washed with PBS. The biofilms were then stained with crystal violet, and the excess stain was eluted with 100% ethanol. Biofilm formation was quantified by measuring the absorbance at 595 nm using a spectrophotometer.

### Measurement of the concentration of GTP

Briefly, 50 mL of *E. flavus* 21-3, *E. coli* BL21 (DE3), and *P. aeruginosa* PAO1 cultures were collected by centrifugation at 3,000 *× g* for 10 minutes. The supernatant was discarded, and the cells were resuspended in 40 mL of PBS. The cells were then sonicated, and the remaining debris was removed by centrifugation at 10,000 *× g* for 10 minutes at 4°C. The supernatant was collected and used to determine the GTP concentration using the GTP ELISA kit (Meimian, China).

### Overexpression of gene *ppk2* in *P. aeruginosa* PAO1 and *E. coli* BL21 (DE3)

To overexpress PPK2 in *P. aeruginosa* PAO1, we selected pUCP18-Gm as the vector, as described in previous studies (71). The PPK2 coding sequence was amplified using the primers listed in Table S4. The PCR products were then recovered and cloned into pUCP18-Gm by homologous recombination. The resulting construct was transformed into *E. coli* for replication, and the final vector was verified by sequencing. Finally, the expression vector was transformed into *P. aeruginosa* PAO1 as previously described (72), and the corresponding assays were performed. The method for overexpressing *ppk2* in *E. coli* BL21 (DE3) is the same as described above, except that the vector is changed to pET28a (+).

### Quantitative real-time PCR (qRT-PCR)

To determine gene transcription in *E. flavus* 21-3, bacterial cells were harvested by centrifugation at 5,000 *× g* for 10 minutes at 4°C. Total RNA was extracted from each sample using Trizol reagent (Solarbio, China). The RNA concentration was measured using a NanoPhotometer NP80 spectrophotometer (Implen, Germany). RNA was then reverse transcribed into cDNA using ReverTra Ace qPCR RT Master Mix with gDNA Remover (TOYOBO, Japan). Gene transcription was assessed by qRT-PCR using a QuantStudio 6 Flex system (Thermo Fisher Scientific, USA) and SYBR Green Realtime PCR Master Mix (TOYOBO, Japan). The PCR conditions were as follows: initial denaturation at 95°C for 1 minute, followed by 40 cycles of denaturation at 95°C for 15 seconds, annealing at 52°C for 15 seconds, and extension at 72°C for 15 seconds. 16S rRNA was used as the internal reference gene. The relative gene expression was calculated using the 2^−ΔΔCt^ method and normalized to the expression of 16S rRNA

### Data analysis

All experiments were repeated three times, and statistical analysis was conducted using data processing software to perform multiple comparisons among the experimental groups. In this study, statistical significance was determined at *P* value and n.s. stands for no significant difference.

## Data Availability

The genome of *E. flavus* 21-3 is in GenBank under accession no. CP032228. Proteomics data have been deposited in PRIDE under accession no. PXD058843. Note: in GenBank, *Qipengyuania flava* 21-3 and *E. flavus* 21-3 are the same strain.

## SUPPLEMENTAL MATERIAL

Supplemental material msystems.00473-25-s0001.pdf Proteomic analysis methods; Figures S1-S5; Tables S1-S4.

ASM does not own the copyrights to Supplemental Material that may be linked to, or accessed through, an article. The authors have granted ASM a non-exclusive, world-wide license to publish the Supplemental Material files. Please contact the corresponding author directly for reuse.
