# Supplementary material for: Light and polyphosphate kinase 2 cooperatively regulate the production of zero-valent sulfur in a deep-sea bacterium
Source: mSystems. 2025 May 16;10(6):e00473-25. doi: 10.1128/msystems.00473-25 (PMC12172448; doi:10.1128/msystems.00473-25)
Supplement: Supplemental material — Proteomic analysis methods; Figures S1-S5; Tables S1-S4. [file msystems.00473-25-s0001.pdf]

# SUPPLEMENTARY INFORMATION

## Light and polyphosphate kinase 2 cooperatively regulate the production of zero-valent sulfur in a deep-sea bacterium

Tianhang Zhang[1,2,3,4], Ruining Cai[1,2,4], Chaomin Sun[1,2,3,4*]

[1]CAS and Shandong Province Key Laboratory of Experimental Marine Biology & Center of Deep Sea Research, Institute of Oceanology, Chinese Academy of Sciences, Qingdao, China.

[2]Laboratory for Marine Biology and Biotechnology, Qingdao Marine Science and Technology Center, Qingdao, China.

[3]College of Earth Science, University of Chinese Academy of Sciences, Beijing, China.

[4]Center of Ocean Mega-Science, Chinese Academy of Sciences, Qingdao, China.

[*] Corresponding author

Chaomin Sun

Tel.: +86 532 82898857; fax: +86 532 82898857.

E-mail address: sunchaomin2020@126.com; sunchaomin@qdio.ac.cn

# SUPPLEMENTARY METHODS

**Proteomic analysis.** To digest proteins, 5 mM dithiothreitol (DTT) was added to reduce the sample, followed by incubation at 65 °C for 30 minutes. Next, 11 mM iodoacetamide was used to alkylate the sample at room temperature for 15 minutes in the dark. The protein sample was then diluted with 100 mM tetraethylammonium bromide (TEAB) until the urea concentration was reduced to less than 2 M. For the first digestion, trypsin was added at a 1:50 trypsin-to-protein mass ratio, followed by a second digestion for 4 hours with a 1:100 trypsin-to-protein mass ratio. After digestion, the sample was desalted using a Strata X C18 SPE column (Phenomenex, USA). Following vacuum drying, the peptide was reconstituted in 0.5 M TEAB and processed according to the TMT kit protocol.

The tryptic peptides were fractionated using high-pH reverse-phase HPLC with an Agilent 300Extend C18 column (4.6 mm ID, 5 μm particles, 250 mm length). The peptides were then combined into 18 fractions and vacuum dried. Tryptic peptides were dissolved in 0.1% formic acid and loaded onto a homemade reverse-phase analytical column (75 μm ID, 15 cm length). The gradient consisted of an increase from 6% to 23% solution B (0.1% formic acid in 98% acetonitrile) over 26 minutes, followed by 23% to 35% over 8 minutes, and then a rise to 80% over 3 minutes. The gradient was held at 80% for 3 minutes, all at a constant flow rate of 0.5 mL/min on an EASY-nLC 1000 UPLC system.

The peptides were analyzed using NSI source tandem mass spectrometry on a Q Exactive$^{TM}$ Plus (Thermo Fisher Scientific, USA) coupled online to UPLC. An electrospray voltage of 2.0 kV was applied, with an *m/z* scan range of 350-1,800 for the full scan. Intact peptides were detected in the Orbitrap with a resolution of 70,000. For MS/MS, normalized collision energy (NCE) was set to 28. Fragment ions were detected in the Orbitrap at a resolution of 17,500. The data-dependent acquisition alternated between 1 MS scan followed by 20 MS/MS scans, with a 15.0 s dynamic exclusion. Automatic gain control (AGC) was set to 5E4, and the fixed first mass was set at 100 *m/z*.

The MaxQuant search engine was used to process the resulting MS/MS data. Tandem mass spectra were searched against the NCBI database, concatenated with a reverse decoy database. Trypsin/P was specified as the cleavage enzyme, allowing for up to two missed cleavages. In both the first and main searches, the mass tolerance for precursor ions was set to 20 ppm and 5 ppm, respectively, while the mass tolerance for fragment ions was set to 0.02 Da. Carbamidomethylation of cysteine (Cys) was specified as a fixed modification, and oxidation of methionine (Met) was set as a variable modification. The false discovery rate (FDR) was adjusted to $< 1\%$, and the minimum peptide score was set to 40.

The peptides sequences were annotated using KEGG (Release 103.0), Pfam (v35.0), COG (v2020), and Uniprot (1-3). The cut-off was set as 1e-20. Protein domains were predicted by NCBI batch web CD-Search Tool (v2020) and further confirmed by the specific Pfam model using HMMer (4, 5).

# SUPPLEMENTARY RESULTS

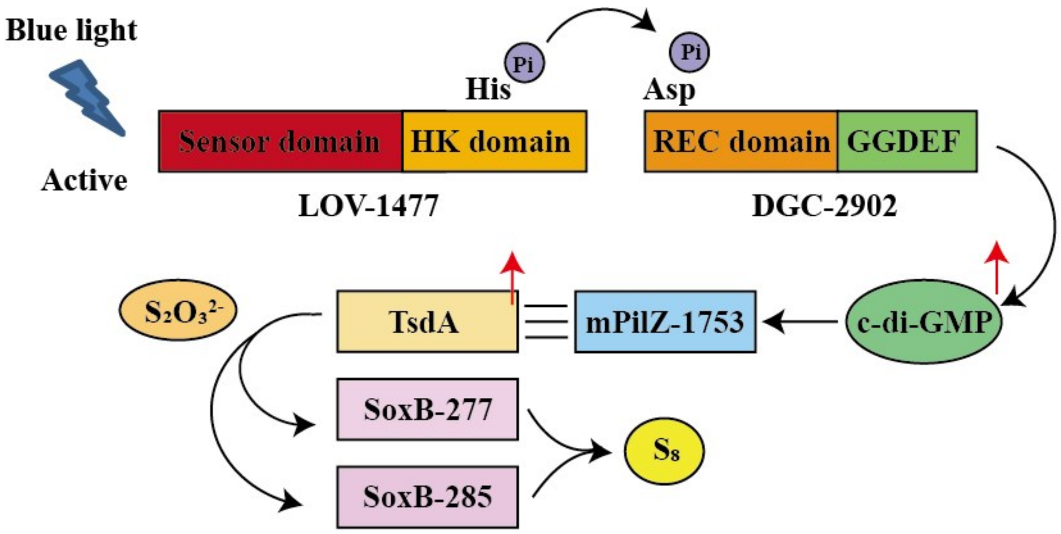


**Supplementary Fig. S1. Proposed model depicting the blue light response and thiosulfate oxidation coupling pathway in *E. flavus* 21-3.** Upon blue light stimulation, autophosphorylation increases at the conserved histidine residue of the LOV histidine kinase LOV-1477. A phosphate group (Pi) is subsequently transferred to the conserved aspartate residue of the response regulator, the diguanylate cyclase DGC-2902. DGC-2902 then synthesizes c-di-GMP as an output response. The c-di-GMP produced binds to mPilZ-1753, which activates thiosulfate dehydrogenase (TsdA) through interaction, leading to enhanced conversion of thiosulfates into tetrathionates. These tetrathionates are subsequently hydrolyzed by thiosulfohydrolases (SoxB-277 and SoxB-285), resulting in the formation of ZVS. The red arrows indicate that blue light stimulation increases the intracellular c-di-GMP concentration and enhances TsdA activity in *E. flavus* 21-3 (6).

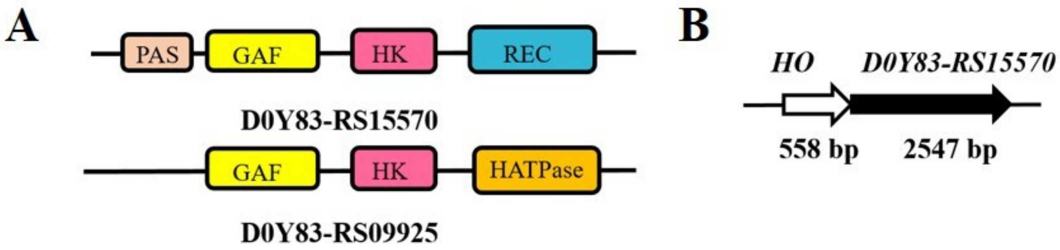


**Supplementary Fig. S2. The predicted protein structures of bacteriophytochromes D0Y83-RS15570 and D0Y83-RS09925.** (A) Domain annotations for D0Y83-RS15570 and D0Y83-RS09925. (B) The gene encoding heme oxygenase (HO) is located upstream of the D0Y83-RS15570 gene. Domain abbreviations: PAS – Per-ARNT-Sim, GAF – cGMP phosphodiesterase/adenylate cyclase/FhlA, HK – histidine kinase, REC – response regulator.

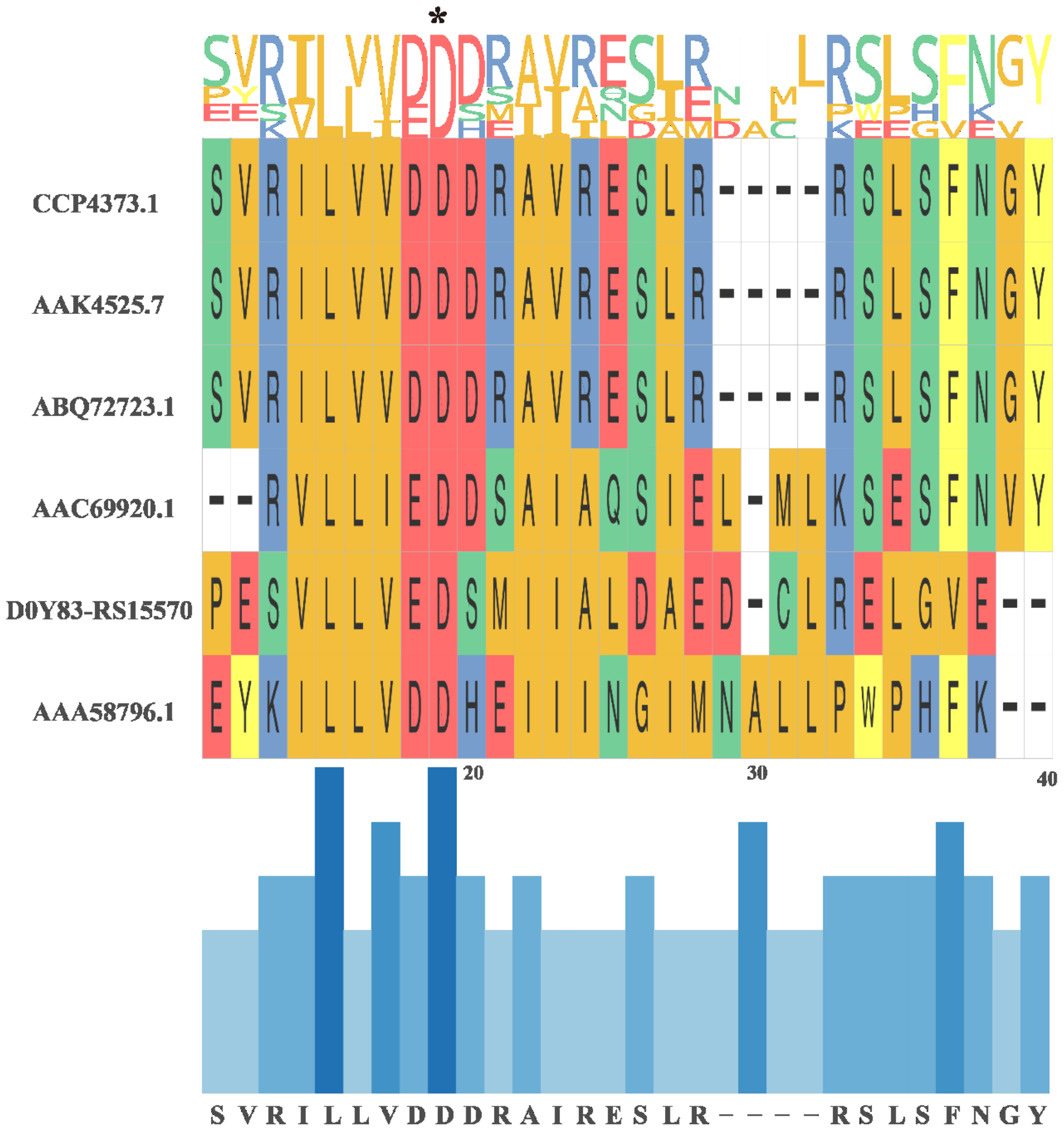


**Supplementary Fig. S3. Sequence alignments of D0Y83-RS15570 and other homologues.** The conserved binding site (Asp in REC domain) was marked with asterisk. The numbers on the left side of the panel indicate the accession numbers of corresponding proteins. The blue bar chart indicates the frequency of base occurrence.

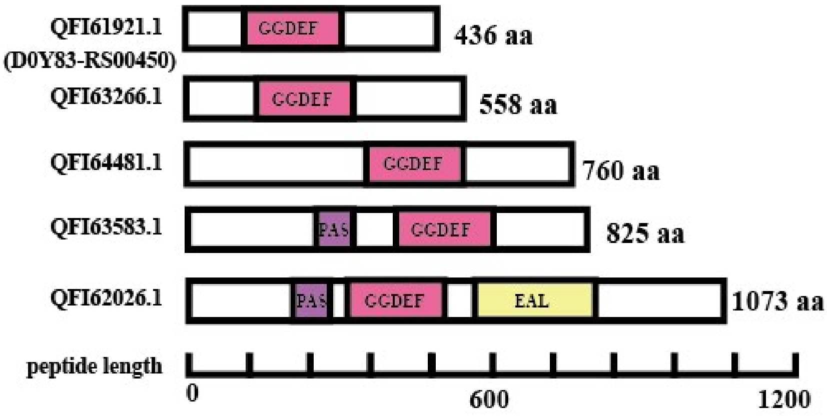


**Supplementary Fig. S4. Domain annotations of five proteins containing GGDEF domain. PAS: Per-ARNT-Sim; REC: response regulator.**

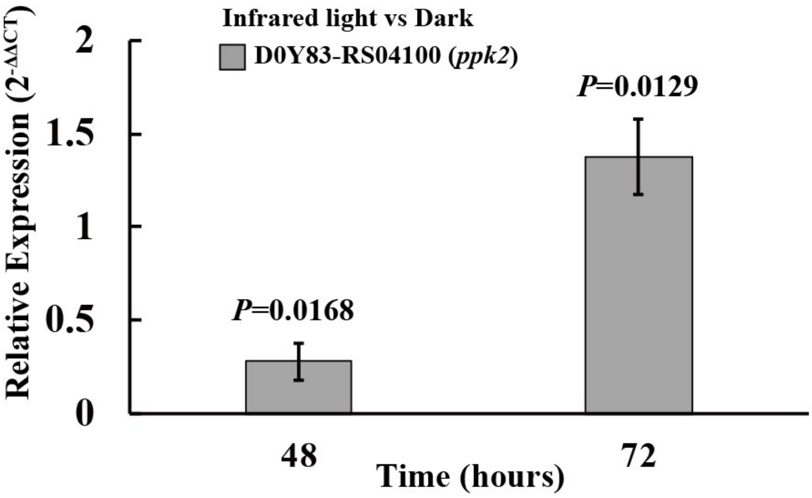


**Supplementary Fig. S5. The relative expression of *ppk2* in *E. flavus* 21-3 was measured after cultivation under infrared light for 48 and 72 hours, compared to cultivation in the dark.**

**Supplementary Table 1. Primers used for cloning the gene *HO* and *bphp-15570***

| Primer name | Sequence (5'-3') |
|---|---|
| 15570-F | AGCAAATGGGTCGCGGATCCATGAACCCGCAGGACGTCAA |
| 15570-R | TGTCGACGGAGCTCGAATTCCCCTGCCGTGGCACTACCCA |
| 15570-pET28a-F | TGGGTAGTGCCACGGCAGGGGAATTCGAGCTCCGTCGACA |
| 15570-pET28a-R | TTGACGTCCTGCGGGTTCATGGATCCGCGACCCATTTGCT |
| HO-F | CGCGTGGATCCCCGGAATTCATGCCGGTATCAAGCGGGAC |
| HO-R | ACGATGCGGCCGCTCGAGTGCCGCCTTACGCATTGCGCTG |
| HO-pGEX4T-1-F | GCGCAATGCGTAAGGCGGCACTCGAGCGGCCGCATCGTGA |
| HO-pGEX4T-1-R | GTCCCGCTTGATACCGGCATGAATTCCGGGGATCCACGCG |

**Supplementary Table 2. Primers used for gene deletion**

| Primer name | Sequence (5'-3') |
|---|---|
| PPK2-leftarm-F | ATGACCATGATTACGAATTCGATCACGTAATCGGTGCGCA |
| PPK2-leftarm-R | ACCTGCAGGTCGCGCGAAGGCGCGCAGCAACTCCCATCGT |
| PPK2-rightarrm-F | ACACAAACGATGGGAGTTGCTGCGCGCCTTCGCGCGACCT |
| PPK2-rightarrm-R | AGGTCGACTCTAGAGGATCCGTGCTCCAGCATCGGCTGCG |
| PPK2-pEX18-F | CGCAGCCGATGCTGGAGCACGGATCCTCTAGAGTCGACCT |
| PPK2-pEX18-R | TGCGCACCGATTACGTGATCGAATTCGTAATCATGGTCAT |
| 15570-leftarm-F | ATGACCATGATTACGAATTCGGGCGCGACACCCGACCAGC |
| 15570-leftarm-R | CGCCGCGCATCGCGAAAGGGCATGCCGCCTTACGCATTGC |
| 15570-rightarm-F | GCAATGCGTAAGGCGGCATGCCCTTTCGCGATGCGCGGCG |
| 15570-rightarm-R | AGGTCGACTCTAGAGGATCCACCGTCGGTGCTCGCGGTCA |
| 15570-pEX18-F | TGACCGCGAGCACCGACGGTGGATCCTCTAGAGTCGACCT |
| 15570-pEX18-R | GCTGGTCGGGTGTCGCGCCCGAATTCGTAATCATGGTCAT |
| pEX18-F | TGAGTTAGCTCACTCATTAGGCACCCCAG |
| pEX18-R | CAGCTGGCGAAAGGGGGATGTGCTGCAAG |

## Supplementary Table 3. Primers used for bacterial two-hybrid assay

| Primer name | Sequence (5'-3') |
|---|---|
| 15570-BTH-F | ACACAGGAAACAGCTATGACATGAACCCGCAGGACGTCAA |
| 15570-BTH-R | TCCAGGCCGCCCGTGGCCTCCCCTGCCGTGGCACTACCCA |
| 15570-pCH363-F | TGGGTAGTGCCACGGCAGGGGAGGCCACGGGCGGCCTGGA |
| 15570-pCH363-R | TTGACGTCCTGCGGGTTCATGTCATAGCTGTTTCCTGTGT |
| pCH363-F | AAGCGGGCAGTGAGCGCAACGCAATTAAT |
| pCH363-R | ATCACGCCGATATTCATGTCGCCGTCGTAG |
| 08245-F | CACAGGAAACAGCTATGACCATGCGCGAACAGATAATGGG |
| 08245-R | GCCTGATGCGATTGCTGCATGCCGCGCGCTTCGCGGCGCC |
| 08245-pKNT25-F | GGCGCCGCGAAGCGCGCGGCATGCAGCAATCGCATCAGGC |
| 08245-pKNT25-R | CCCATTATCTGTTCGCGCATGGTCATAGCTGTTTCCTGTG |
| 00450-F | CACAGGAAACAGCTATGACCATGCCCGCCAAACTCCCCGA |
| 00450-R | GCCTGATGCGATTGCTGCATGGCGCTGAGGATGCAGTTGC |
| 00450-pKNT25-F | GCAACTGCATCCTCAGCGCCATGCAGCAATCGCATCAGGC |
| 00450-pKNT25-R | TCGGGGAGTTTGGCGGGCATGGTCATAGCTGTTTCCTGTG |
| 01080-F | CACAGGAAACAGCTATGACCATGGCTGAACACGCCACTCA |
| 01080-R | GCCTGATGCGATTGCTGCATGCCGGCCTCGCGCCCGCGAC |
| 01080-pKNT25-F | GTCGCGGGCGCGAGGCCGGCATGCAGCAATCGCATCAGGC |
| 01080-pKNT25-R | TGAGTGGCGTGTTCAGCCATGGTCATAGCTGTTTCCTGTG |
| 10085-F | CACAGGAAACAGCTATGACCATGATGCACCCTGGCCTGCG |
| 10085-R | GCCTGATGCGATTGCTGCATGCCGGCGATCTTGACCTGCG |
| 10085-pKNT25-F | CGCAGGTCAAGATCGCCGGCATGCAGCAATCGCATCAGGC |
| 10085-pKNT25-R | CGCAGGCCAGGGTGCATCATGGTCATAGCTGTTTCCTGTG |
| 15350-F | CACAGGAAACAGCTATGACCGGATTTTGCCGAGGCAACTG |
| 15350-R | GCCTGATGCGATTGCTGCATGCGAGCGCTGTGCGGGGCTC |
| 15350-pKNT25-F | GAGCCCCGCACAGCGCTCGCATGCAGCAATCGCATCAGGC |
| 15350-pKNT25-R | CAGTTGCCTCGGCAAAATCCGGTCATAGCTGTTTCCTGTG |
| 14455-F | CACAGGAAACAGCTATGACCGTGGATGACGTTCCCGAGAG |

**Supplementary Table 3. Primers used for bacterial two-hybrid assay (continued)**

| **Primer name** | **Sequence** (5'-3') |
|---|---|
| 14455-R | GCCTGATGCGATTGCTGCATTTGTCGCTCCAGAAGGACTT |
| 14455-pKNT25-F | AAGTCCTTCTGGAGCGACAAATGCAGCAATCGCATCAGGC |
| 14455-pKNT25-R | CTCTCGGGAACGTCATCCACGGTCATAGCTGTTTCCTGTG |
| pKNT25-F | AGCGCAACGCAATTAATGTGAGTTAGCTC |
| pKNT25-R | GACCAGGCGGAACATCAATGTGGCGTTTT |

**Supplementary Table 4. Primers used for overexpression of gene *ppk2* in *P. aeruginosa* PAO1 and *E. coli* BL21 (DE3)**

| **Primer name** | **Sequence (5'-3')** |
|---|---|
| PPK2-PAO1-F | CGACGGCCAGTGCCAAGCTTATGAAACGCAAGCAATATGA |
| PPK2-PAO1-R | AGCTCGGTACCCGGGGATCCTCCCAGGGGATAATTAGCAA |
| PPK2-pucp18-F | TTGCTAATTATCCCCTGGGAGGATCCCCGGGTACCGAGCT |
| PPK2-pucp18-R | TCATATTGCTTGCGTTTCATAAGCTTGGCACTGGCCGTCG |
| pUCP18-Gm-F | TATTACGCCAGCTGGCGAAAGGGGGATGT |
| pUCP18-Gm-R | CCGCGCGTTGGCCGATTCATTAATGCAGC |
| PPK2-F | AGCAAATGGGTCGCGGATCCAAACGCAAGCAATATGAGAT |
| PPK2-pET28a-R | ATCTCATATTGCTTGCGTTTGGATCCGCGACCCATTTGCT |
| PPK2-pET28a-F | TTGCTAATTATCCCCTGGGAAAGCTTGCGGCCGCACTCGA |
| PPK2-R | TCGAGTGCGGCCGCAAGCTTTCCCAGGGGATAATTAGCAA |

# REFERENCES


1. **Kanehisa M, Sato Y, Morishima K**. 2016. BlastKOALA and GhostKOALA: KEGG Tools for functional characterization of genome and metagenome sequences. *J Mol Biol* 428:726-731.
2. **Galperin MY, Wolf YI, Makarova KS, Alvarez RV, Landsman D, Koonin EV**. 2021. COG database update: focus on microbial diversity, model organisms, and widespread pathogens. *Nucleic Acids Res* 49:274-281.
3. **Mistry J, Chuguransky S, Williams L, Qureshi M, Salazar GA, Sonnhammer ELL, Tosatto SCE, Paladin L, Raj S, Richardson LJ, Finn RD, Bateman A**. 2021. Pfam: The protein families database in 2021. *Nucleic Acids Res* 49:412-419.
4. **Mistry J, Finn RD, Eddy SR, Bateman A, Punta M**. 2013. Challenges in homology search: HMMER3 and convergent evolution of coiled-coil regions. *Nucleic Acids Res* 41.
5. **Lu SN, Wang JY, Chitsaz F, Derbyshire MK, Geer RC, Gonzales NR, Gwadz M, Hurwitz DI, Marchler GH, Song JS, Thanki N, Yamashita RA, Yang MZ, Zhang DC, Zheng CJ, Lanczycki CJ, Marchler-Bauer A**. 2020. CDD/SPARCLE: the conserved domain database in 2020. *Nucleic Acids Res* 48:265-268.
6. **Cai RN, He WY, Zhang J, Liu R, Yin ZY, Zhang X, Sun CM**. 2023. Blue light promotes zero-valent sulfur production in a deep-sea bacterium. *EMBO J* 42.
